# Carprofen-induced depletion of proton motive force reverses TetK-mediated doxycycline resistance in methicillin-resistant *Staphylococcus pseudintermedius*

**DOI:** 10.1038/s41598-019-54091-4

**Published:** 2019-11-28

**Authors:** Zofia Magnowska, Bimal Jana, Rikke Prejh Brochmann, Andrew Hesketh, Rene Lametsch, Cristian De Gobba, Luca Guardabassi

**Affiliations:** 1 Department of Veterinary and Animal Sciences, Faculty of Health and Medical Sciences, University of Copenhagen, Frederiksberg, Denmark; 2 Department of Biochemistry and Cambridge Systems Biology Centre, University of Cambridge, Cambridge, United Kingdom; 3 School of Pharmacy and Biomolecular Sciences, University of Brighton, Brighton, United Kingdom; 4 Department of Food Science, Faculty of Sciences, University of Copenhagen, Frederiksberg, Denmark; 5 Department of Pathobiology and Population Sciences, The Royal Veterinary College, Hatfield, United Kingdom

**Keywords:** Antimicrobial resistance, Systems biology

## Abstract

We previously showed that doxycycline (DOX) and carprofen (CPF), a veterinary non-steroidal anti-inflammatory drug, have synergistic antimicrobial activity against methicillin-resistant *Staphylococcus pseudintermedius* (MRSP) carrying the tetracycline resistance determinant TetK. To elucidate the molecular mechanism of this synergy, we investigated the effects of the two drugs, individually and in combination, using a comprehensive approach including RNA sequencing, two-dimensional differential in-gel electrophoresis, macromolecule biosynthesis assays and fluorescence spectroscopy. Exposure of TetK-positive MRSP to CPF alone resulted in upregulation of pathways that generate ATP and NADH, and promote the proton gradient. We showed that CPF is a proton carrier that dissipates the electrochemical potential of the membrane. In the presence of both CPF and DOX, the energy compensation strategy was attenuated by downregulation of all the processes involved, such as citric acid cycle, oxidative phosphorylation and ATP-providing arginine deiminase pathway. Furthermore, protein biosynthesis inhibition increased from 20% under DOX exposure alone to 75% upon simultaneous exposure to CPF. We conclude that synergistic interaction of the drugs restores DOX susceptibility in MRSP by compromising proton-motive-force-dependent TetK-mediated efflux of the antibiotic. MRSP is unable to counterbalance CPF-mediated PMF depletion by cellular metabolic adaptations, resulting in intracellular accumulation of DOX and inhibition of protein biosynthesis.

## Introduction

Staphylococci are common colonizers of the skin and mucosae in both humans and animals, and cause a wide range of opportunistic infections such as urinary tract, skin and soft-tissue infections, otitis, endocarditis, osteomyelitis and sepsis. Historically, β-lactamase-insensitive β-lactam antibiotics exhibited good activity against staphylococci but the emergence of methicillin resistance has made them ineffective. Methicillin resistance is mediated by the *mecA* gene, which encodes a modified penicillin-binding protein PBP2a with low affinity for all conventional β-lactams^1^. The global prevalence of methicillin-resistant *Staphylococcus aureus* (MRSA) and methicillin-resistant *Staphylococcus pseudintermedius* (MRSP) is increasing in both human and veterinary medicine^2^. Some epidemic MRSP clones such as sequence type (ST)71 are virtually resistant to all antibiotics available in veterinary medicine^3^.

Combination therapy is one of the possible strategies to manage infections caused by multidrug-resistant bacteria. In a previous study^4^, we identified a strong synergy between doxycycline (DOX), a tetracycline antibiotic that inhibits bacterial protein synthesis by binding to the 30 S ribosomal subunit, and carprofen (CPF), a veterinary non-steroidal anti-inflammatory drug (NSAID) that inhibits cyclooxygenase activity in eukaryotic cells. CPF was previously shown to have antibacterial activity against *Escherichia coli* by inhibiting DNA polymerase III β subunit (also known as the sliding clamp), which plays an essential role in DNA replication and repair^5^. We showed that co-exposure to CPF restored susceptibility to DOX in DOX-resistant MRSP ST71 containing *tetK*, a tetracycline resistance gene encoding a drug-specific efflux pump^6^. No synergy was observed for other MRSP strains where DOX resistance was mediated by other mechanisms (e.g. ribosomal protection mediated by *tetM*).

The aim of this study was to elucidate the molecular mechanism of synergy between DOX and CPF. The effects of the two drugs, individually and in combination, were investigated in DOX-resistant MRSP ST71 using a comprehensive systems biology approach consisting of RNA sequencing (RNA-seq), two-dimensional differential in-gel electrophoresis (2D DIGE), macromolecule biosynthesis assays and fluorescence spectroscopy.

## Results

### Transcriptomic and proteomic changes following exposure to CPF and/or DOX

RNA-seq and 2D DIGE were performed to analyse the effects of the two drugs alone and in combination on transcript and protein abundance, respectively, in the TetK-positive MRSP ST71 model strain E104. Bacterial cultures were sampled after 30 min exposure to analyse the early transcriptomic response and after 90 min to examine both transcriptomic and proteomic responses. Supplementary Figure S1 summarise experimental plan for the expression study. Transcript abundance data for 2515 genes (for both time points, passing the count filter) and protein abundance data for 516 protein spots (passing the Biological Variation Analysis (BVA) gel to gel matching of spots) were used for gene expression analysis.

Substantial changes were observed in global gene expression when MRSP E104 was exposed to DOX and CPF in combination as compared to individual drug exposure. The numbers of significantly regulated transcripts (±2.0-fold change (FC), p < 0.05) and proteins (±1.5-FC, p < 0.05) for CPF and DOX separately and in combination are summarised in Table 1. DOX alone and in combination with CPF caused massive changes in gene expression with transcription of a total of 1507 and 1594 different genes changing when considering both the 30 and 90 min time points, respectively. In comparison, CPF alone changed expression of only 310 unique genes in total. The same ratio of proteins regulated by DOX and CPF separately (5:1) was observed in the proteome analysis (184/36). Fewer significant changes in transcript abundance were detected after 30 min than after 90 min. Supplementary Table S1 presents a list of all genes differentially regulated by CPF and/or DOX.

**Table 1:** Summary of gene regulation results associated with exposure to doxycycline (DOX) and/or carprofen (CPF) and their synergistic interactions.

	Regulations:	DOX	CPF	DOX-CPF	Synergistic interactions
Transcriptome 30 min	Up	656	22	601	11
	Down	464	8	457	14
	**Sum**	**1120**	**30**	**1058**	**25**
Transcriptome 90 min	Up	638	156	744	252
	Down	590	143	696	228
	**Sum**	**1228**	**299**	**1440**	**480**
Proteome 90 min	Up	74	15	102	32
	Down	110	21	139	28
	**Sum**	**184**	**36**	**241**	**60**

### CPF regulates metabolic pathways associated with cellular energetics

With very few exceptions, we did not detect any major transcriptomic changes after 30 min exposure to CPF. The following paragraphs mainly refer to the transcriptomic data obtained following 90 min exposure, when alterations in the transcriptome were more frequent than those observed in the proteome. The bacitracin ABC transporter permease *bceB* and bacitracin ABC transporter ATP-binding protein *bceA* were the genes most intensely induced by CPF, while tryptophan biosynthesis was the most notably induced pathway. All genes encoding enzymes catalysing synthesis of tryptophan from chorismate (*trp ABFCDGE*, *trpA* detected also at the protein level) were strongly upregulated after exposure to CPF. As highlighted in the following paragraphs, cellular energetics was the cellular function most consistently and strongly impacted by CPF. Table 2 summarizes all the regulated proteins mentioned in the omics description.

**Table 2:** Summary list of the genes described in the text presented with significant regulation results associated with exposure to CPF and CAR/DOX synergistic interactions. T - transcriptomic and P - proteomics results. Complete gene expression data including adjusted p-values are listed in the Supplementary Table S1.

Gene ID	Product	Name	CPF_log_2_FC			Interactions_log_2_FC			Function
Time point			T	T	P	T	T	P	
			30	90	90	30	90	90	
**Central metabolism**									
UH47_06750	PTS system glucose-specific transporter subunit			**−1**					glycolysis
UH47_06270	PTS system glucose-specific transporter subunit			**−1.2**					glycolysis
UH47_12920	glyceraldehyde-3-phosphate dehydrogenase			**2.1**			**−1.5**		glycolysis/gluconeogenesis
UH47_11065	phosphoglyceromutase	*gpmI*				**1.3**			glycolysis/gluconeogenesis
UH47_11070	triosephosphate isomerase	*tpi*			**−1.2**	**1.5**		**1.3**	glycolysis/gluconeogenesis
UH47_11075	phosphoglycerate kinase	*pgk*				**1.4**		**1.1**	glycolysis/gluconeogenesis
UH47_11085	central glycolytic genes regulator	*cggR*				**1**			glycolysis
UH47_12460	pyruvate dehydrogenase						**−1.1**		glycolysis
UH47_01940	glucarate transporter	*gudP*		**1**			**−1.2**		alternative carbon source
UH47_02965	PTS alpha-glucoside transporter subunit IIBC			**1.3**					alternative carbon source
UH47_02580	melibiose:sodium symporter	*melB*		**1.1**			**−1.9**		alternative carbon source
UH47_02745	trehalose-6-phosphate hydrolase	*treC*		**1.7**			**−2.4**		alternative carbon source
UH47_02750	trehalose permease IIC protein			**2.6**			**−3.1**		alternative carbon source
UH47_07415	gluconokinase	*gntK*		**1.6**			**−1.4**		alternative carbon source
UH47_12030	glycerol-3-phosphate dehydrogenase	*glpA*	**2.2**	**2.5**			**−2**		alternative carbon source
UH47_12040	glycerol transporter	*glpF*	**1.8**	**2.2**			**−1.9**		alternative carbon source
UH47_12035	glycerol kinase	*glpK*	**1.3**	**2.1**			**−2**		alternative carbon source
UH47_00550	C4-dicarboxylate ABC transporter	*sdcS*		**1.2**					alternative carbon source
UH47_10320	acetyl-CoA synthetase	*acs*		**1.7**			**−2**		alternative carbon source
UH47_10480	acetate kinase	*ackA*			**1**			**−0.8**	alternat. carbon source/ ferment.
UH47_10315	acetoin dehydrogenase	*acuA*		**1.8**			**−1.2**		alternat. carbon source/ ferment.
UH47_10310	acetoin utilization protein AcuC	*acuC*		**1.0**					alternat. carbon source/ ferment.
UH47_06315	lactate permease	*lctP*		**1.2**			**−1.7**		alternat. carbon source/ ferment.
UH47_07720	pyruvate oxidase	*poxL*		**1.2**			**−1.4**		alternat. carbon source/ ferment.
UH47_12345	acyl CoA:acetate/3-ketoacid CoA transferase	*atoD*		**3**			**−2.3**		alternat. carbon source/ ferment.
UH47_01900	pyruvate formate lyase-activating protein	*pflA*		**−3**			**2.9**		fermentation
UH47_06300	alcohol dehydrogenase	*adh*		**−2.2**			**2.6**		fermentation
UH47_12335	butyryl-CoA dehydrogenase			**−1.5**			**1.5**		fermentation
UH47_05215	succinyl-CoA synthetase subsunit alpha	*sucD*			**0.7**		**−1.6**	**−0.7**	TCA cycle
UH47_05220	succinyl-CoA synthetase subunit beta	*sucC*					**−1.7**		TCA cycle
UH47_05745	succinate dehydrogenase	*sdhB*					**−1.6**		TCA cycle
UH47_05750	succinate dehydrogenase	*sdhA*					**−1.9**		TCA cycle
UH47_06505	malate:quinone oxidoreductase	*mqo*						**−0.8**	TCA cycle
UH47_06710	aconitate hydratase	*acnA*		**1.6**			**−2.4**	**−2**	TCA cycle
UH47_06935	dihydrolipoamide succinyltransferase	*sucB*		**1.2**	**0.8**		**−1.7**	**−0.6**	TCA cycle
UH47_06940	2-oxoglutarate dehydrogenase	*sucA*		**1.3**			**−1.9**		TCA cycle
UH47_10555	citrate synthase	*gltA*		**2**			**−3.1**		TCA cycle
UH47_10560	isocitrate dehydrogenase	*icd*		**2**			**−3.4**		TCA cycle
UH47_11305	fumarate hydratase	*fum*		**1.1**			**−1.9**		TCA cycle
UH47_05755	succinate dehydrogenase	*sdhC*		**1**			**−2.1**		TCA cycle/electron transport
**Respiration**									
UH47_10780	ATP synthase F0F1 subunit gamma						**−1**		ATP synthase
UH47_10785	ATP F0F1 synthase subunit alpha						**−1.1**		ATP synthase
UH47_10790	ATP synthase F0F1 subunit delta						**−1.1**		ATP synthase
UH47_09665	2-succinyl-6-hydroxy-2, 4-cyclohexadiene-1-carboxylate synthase	*menH*					**−1**		electron transfer chain
UH47_09670	dihydroxynaphthoic acid synthetase	*menB*					**−1**		electron transfer chain
UH47_06375	FMN-dependent NADH:quinone azoreductase	*acpD*	**1.2**	**1.6**					electron transfer chain
UH47_09945	cytochrome bd menaquinol oxidase	*cydA*		**−2.5**			**2.6**		electron transfer chain
UH47_09950	cytochrome bd menaquinol oxidase	*cydB*		**−2.7**			**2.8**		electron transfer chain
UH47_01680	nitrate transporter NarT	*narT*		**2**					nitrate respiration
UH47_01705	nitrate reductase	*narJ*		**−2.7**			**2.7**		nitrate respiration
UH47_01710	nitrate reductase	*narH*		**−2.4**			**2.4**		nitrate respiration
UH47_01745	nitrite reductase	*nirD*		**−2.5**					nitrate respiration
UH47_08305	formate/nitrite transporter			**−1.4**			**2**		nitrate respiration
UH47_12325	nitrate ABC transporter substrate-binding protein			**−1.1**			**1**		nitrate respiration
**Amino acid metabolism**									
UH47_08215	octopine dehydrogenase	*odh*		**1.1**			**−1.1**		arginine
UH47_07780	glutamate-1-semialdehyde aminotransferase	*hemL*					**−1**		arginine biosynthesis
UH47_02470	ornithine carbamoyltransferase	*arcB*		**3**		**−1.1**	**−2.9**		arginine utilisation
UH47_06040	arginine deiminase	*arcA*		**1.8**			**−1.2**		arginine utilisation
UH47_06025	carbamate kinase	*arcC*						**−1.1**	arginine utilisation
UH47_00790	ornithine cyclodeaminase			**1.3**			**−1.1**		arginine/proline
UH47_08665	proline dehydrogenase			**2.2**			**−2.4**		arginine/proline
UH47_09190	glutamate dehydrogenase	*gdhA*		**1.1**			**−1.3**		arginine/proline
UH47_09195	ornithine–oxo-acid aminotransferase	*rocD*		**1.5**			**−2.1**		arginine/proline
UH47_09200	1-pyrroline-5-carboxylate dehydrogenase			**1.8**			**−2.5**		arginine/proline
UH47_11985	glutamine synthetase	*glnA*		**−1.2**	**−1**			**0.99**	glutamine biosynthesis
UH47_02010	tryptophan synthase subunit alpha	*trpA*		**3.6**	**1**		**−4.8**	**−1.2**	tryptophan biosynthesis
UH47_02015	tryptophan synthase subunit beta	*trpB*		**3.7**			**−5.4**		tryptophan biosynthesis
UH47_02020	N-(5′-phosphoribosyl)anthranilate isomerase	*trpF*		**3.7**			**−5**		tryptophan biosynthesis
UH47_02025	indole-3-glycerol phosphate synthase	*trpC*		**3.7**			**−4.4**		tryptophan biosynthesis
UH47_02030	anthranilate phosphoribosyltransferase	*trpD*		**3.8**			**−4.5**		tryptophan biosynthesis
UH47_02035	anthranilate synthase subunit II	*trpG*		**3.7**			**−4.9**		tryptophan biosynthesis
UH47_02040	anthranilate synthase component I	*trpE*		**2.8**			**−3.8**		tryptophan biosynthesis
UH47_10825	serine hydroxymethyltransferase	*glyA*					**−1.7**	**0.7**	glicyne/serine
**Lipid metaboism**									
UH47_02215	triacylglycerol lipase	*lip*		**1.5**			**−1.3**		lipid degradation
UH47_12350	long-chain fatty acid–CoA ligase	*acsL*		**4.1**			**−3.4**		lipid degradation
**Protein synthesis**									
UH47_11105	Clp protease	*clpP*			**0.7**			**−0.7**	folding
UH47_01445	ribosome-associated translation inhibitor	*raiA*			**1.1**			**−1.7**	translation
UH47_00215	elongation factor Tu	*tuf*						**−1.6**	translation
UH47_00165	50 S ribosomal protein L1							**0.8**	translation
UH47_00170	50 S ribosomal protein L10	*rplJ*						**2.1**	translation
UH47_00175	50 S ribosomal protein L7/L12	*rplL*						**0.8**	translation
UH47_03310	30 S ribosomal protein S6	*rpsF*						**0.8**	translation
UH47_05280	50 S ribosomal protein L19							**0.7**	translation
UH47_07890	30 S ribosomal protein S5							**0.8**	translation
UH47_11830	50 S ribosomal protein L25	*rplY*						**1.5**	translation
UH47_03525	seryl-tRNA synthetase							**0.7**	translation
UH47_05175	30 S ribosomal protein S2	*rpsB*			**0.6**			**−0.7**	translation
UH47_04780	alanine–tRNA ligase	*alaS*						**1.1**	translation
UH47_04960	valine–tRNA ligase	*valS*						**1**	translation
UH47_11775	methionine–tRNA ligase				**−0.6**			**0.9**	translation
**Other proteins**									
UH47_09860	phosphoribosylformylglycinamidine synthase					**1**			purine metabolism
UH47_13130	tetracycline resistance protein	*tetK*		**−1.0**			**1.1**		resistance
UH47_08515	TetR family transcriptional regulator			**−1.1**			**1.2**		resistance
UH47_00970	bacitracin ABC transporter ATP-binding protein	*bceA*	**1.4**	**4.1**		**−1.5**	**−3.8**		resistance
UH47_00975	bacitracin ABC transporter permease	*bceB*	**1.5**	**4.3**		**−1.6**	**−4**		resistance
UH47_03290	catalase	*katE*		**1.6**			**−1.2**		stress response
UH47_10565	PhoP family transcriptional regulator	*phoB*					**−1.2**	**−1**	phosphate starvation regulator
UH47_00025	pyridoxal biosynthesis protein	*pdxS*					**−1.7**	**0.9**	cofactor biosynthesis

#### Central carbon metabolism

Altogether, the expression of 32 genes involved in central carbon metabolism was significantly altered (at both transcript and protein level). Substantial and consistent alterations were observed for carbohydrate uptake systems. Glucose uptake by the glucose-specific transporter (UH47_06270, UH47_06750) was downregulated, whereas expression of numerous alternative carbohydrate carbon source uptake and activation proteins were triggered, i.e. trehalose (permease, UH47_02750; 6-phosphate hydrolase, *treC*), glucoside (specific transporter, UH47_02965), glucarate (transporter, *gudP*), glycerol (transporter, *glpF*; kinase, *glpK*; dehydrogenase, *glpA*), melibiose (transporter, *melB*) and gluconate (gluconokinase, *gntK*). All alternative carbon source genes were regulated only after 90 min of exposure to CPF with the exception of those encoding lactate permease and glycerol uptake and utilisation enzymes. Only limited changes were observed in the expression of genes with functions in glycolysis/gluconeogenesis.

Bacterial fermentation pathways regenerate NAD^+^ reduced during glycolysis in case of deactivated respiration processes. We observed downregulation of ethanol (pyruvate-formate lyase, *pflA*; alcohol dehydrogenase, *adh*) and butyrate (butyryl-CoA dehydrogenase, UH47_12335) fermentation. Moreover, genes involved in uptake and utilisation of lactate (permease, *lctP*), acetate (acetyl-CoA synthetase, *acs*; pyruvate oxidase, *poxL* and on protein level acetate kinase, *ackA*) and acetonin (dehydrogenase *acuA*; utilisation protein *acuC*, acyl CoA-transferase, *atoD*) were upregulated.

Exposure to CPF consistently induced gene expression for nearly all reactions of the TCA cycle (citrate synthase, *gltA*; aconitate hydratase, *acnA*; isocitrate dehydrogenase, *icd*; 2-oxoglutarate dehydrogenase, *sucA*; dihydrolipoamide succinyltransferase, *sucB*; succinate dehydrogenase*, sdhC*; fumarate hydratase, *fum*). Upregulation of succinyl-CoA synthetase subsunit alpha (*sucD*) and *sucB* was also observed at the protein level. Only conversion between malate and oxaloacetale was not affected. In addition to the glyceraldehyde 3-phosphate dehydrogenase (UH47_12920) enzyme upregulated in glycolysis, two of the reactions upregulated in the TCA cycle increase the cellular pool of NADH, one provides FADH_2_ and the other stores energy in the form of GTP (convertible to ATP). Moreover, aerobic C4-dicarboxylate transporter (*sdcS*) that supplies the TCA cycle with succinate, fumurate and malate was also upregulated. The corresponding anaerobic version of this transporter (*dcuA*) was downregulated.

#### Respiratory gene expression

BLAST analysis modelled on *S. aureus* indicated that *S. pseudintermedius* contains a complete electron transport system and is capable of both aerobic and anaerobic respiration. The electron transport chain consists of NADH dehydrogenase (complex I), succinate dehydrogenase (complex II) that probably, as predicted in *S. aureus*^7^, transfers electrons to the lipophilic isoprenoid electron carrier menaquinone. Menaquinone passes the electrons on the terminal cytochromes of complex IV where O_2_ is reduced to H_2_O. Proton motive force (PMF) generated at complex I and IV drives the F_0_F_1_ ATP synthase. *S. pseudintermedius* can also respire anaerobically using nitrate and nitrite reductase complexes that reduce NO^3−^ to NO^2−^ and NH_3_.

Despite the observations above, which predict an increase in cellular NADH and FADH_2_ pools, we did not observe a marked alteration in the expression of genes encoding the respiratory chain. The FMN-dependent NADH:quinone azoreductase *acpD*, one of 15 genes encoding the NADH dehydrogenase complex, was however significantly upregulated. The AcpD protein plays an important role in removing electrons from NADH and passing them to the electron transfer chain. Cytochrome b (*sdhC*), one of three succinate dehydrogenase subunits from complex II was also significantly upregulated in response to CPF, although this complex participates only in electron passage without proton pumping. Expression of all 4 subunits (*qoxABCD*) of terminal oxidase was not significantly affected by CPF. In common with *S. aureus*^8^*, S. pseudintermedius* has a branched electron transfer chain. In addition to the aforementioned constituents of complex IV, which are required under aerobic conditions, it also possesses the cytochrome bd menaquinol oxidase *cydAB* that is expressed under microaerobic conditions. This oxidase was significantly downregulated in the presence of CPF.

Nitrate and nitrite serve as alternatives to oxygen terminal electron acceptors. We found that both nitrate (*narHJ*) and nitrite (*nirD*) reductase components were downregulated by CPF as well as nitrate ABC transporter substrate-binding protein (UH47_12325) and formate/nitrite transporter nitrate (UH47_08305) transporters. The only exception was upregulation of the nitrate transporter (*narT*). No genes for molybdenum cofactor (Moco) biosynthesis, required for the reduction of nitrate to nitrite^9^, were identified as being differentially regulated, and the entire F_0_F_1_ ATP synthase complex (*atpBEFHAGDC*) and ATP-binding protein (*atpI*) were similarly unaffected by CPF in comparison to the control culture.

#### Arginine metabolism

As summarised in Table 2, consistent upregulation was detected for most of the genes encoding enzymatic steps of conversion between 2-oxoglutarate precursor and arginine via glutamate, glutamate 5-semialdehyde, 1-pyrroline-5-carboxylate, ornithine and citruline (including branches from 1-pyrroline-5-carboxylate to proline by proline dehydrogenase (UH47_08665) and from ornithine to proline by ornithine cyclodeaminase (UH47_00790). Conversion between glutamate and glutamine (by glutamine synthetase *glnA*) was downregulated at both RNA and protein levels. Octopine degradation by octopine dehydrogenase (*odh*) was upregulated. This reaction provides arginine, and in addition one molecule of pyruvate and NADH. Catabolic reactions of arginine, catalysed by ornithine carbamoyltransferase (*arcB*) and arginine deiminase (*arcA*), were also upregulated. This pathway, called the arginine deiminase pathway (ADI), results in ATP formation.

#### Lipid catabolism

The first step of triacylglycerides degradation is the hydrolysis of the ester bonds for breakdown to glycerol and three fatty acids. Exposure to CPF resulted in upregulation of triacylglycerol lipase (*lip*) that catalyses this reaction. One of the top upregulated genes (FC = 16.4) was the long-chain fatty acid-CoA ligase *acsL*, which activates the breakdown of complex fatty acids. This is the pre-step reaction of β-oxidation that abundantly provides acetyl-CoA to the TCA cycle.

### CPF exposure increases the rate of cellular ethidium bromide (EtBr) incorporation

**Figure 1:**
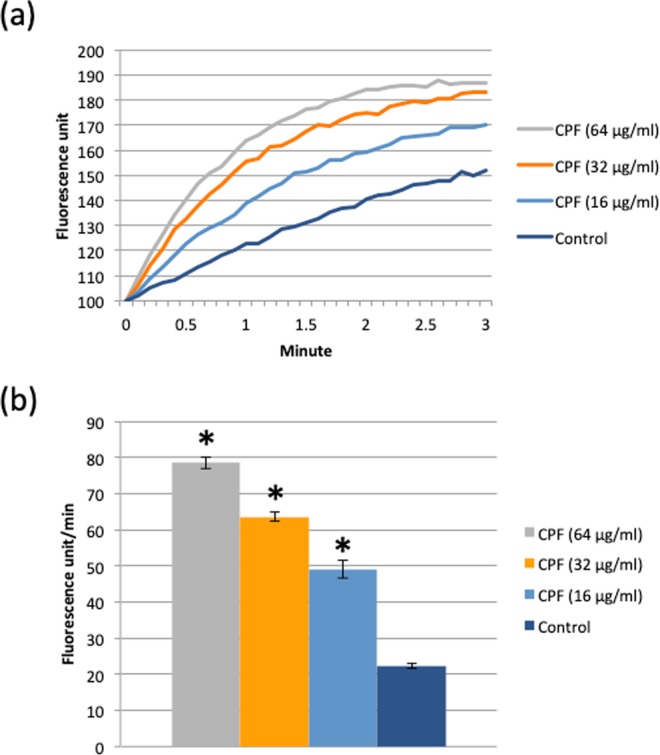
EtBr incorporation rate increases with increasing concentrations of CPF. (**a**) EtBr fluorescence spectra of CPF-exposed and control cells during 3 min exposure. (**b**) Average EtBr fluorescence increment following exposure to different concentrations of CPF. A two-tailed t-test between control and samples was performed, significant difference with p < 0.05 is highlighted by asterisk.

Integrated analysis of the transcriptome and proteome data suggests CPF causes changes in NADH and ATP generation. This and the fact that synergy is specific only for tetK-mediated resistance suggests that CPF affects membrane energetics. To evaluate the effects of CPF on the bacterial membrane and its PMF energy, the rate of EtBr incorporation was studied in MRSP E104. Under normal conditions, PMF-dependent efflux pumps maintain low intracellular concentrations of EtBr^10,11^. Any factors contributing to a loss of membrane integrity, PMF energy or efflux pump functions cause a higher rate of intracellular EtBr accumulation, resulting in an increase of EtBr fluorescence. EtBr fluorescence increased with increasing CPF concentrations (Fig. 1a) and the initial rate of increment for the first 30 seconds was approximately 4 times higher upon exposure to 64 µg/ml of CPF than in the control (Fig. 1b).

### CPF dissipates the cytoplasmic membrane PMF

**Figure 2:**
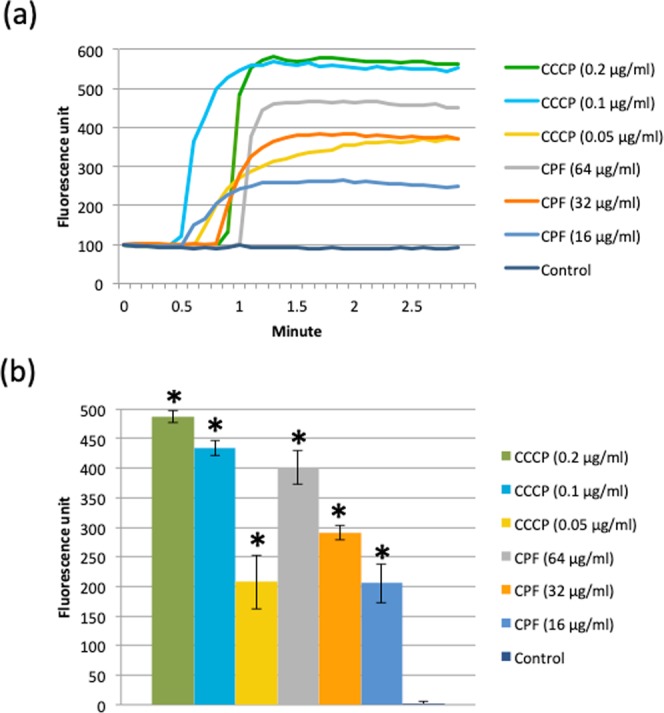
CPF treatment dissipates the PMF of E104. (**a**) DiSC3(5) fluorescence spectra and the effects of individual CPF or CCCP treatments on DiSC_3_(5) fluorescence. (**b**) Average DiSC_3_(5) fluorescence increases upon exposure to CPF or CCCP. A two-tailed t-test between control and samples was performed, significant difference with p < 0.05 is highlighted by asterisk.

We used a membrane-specific fluorescent dye, 3,3′-Dipropylthiadicarbocyanine iodide (DiSC_3_(5)), to determine the effect of CPF on PMF. PMF or protonic potential difference (Δp) of bacterial membrane comprise of two parts, electrochemical potential (ΔΨ) and pH difference between inside and outside (ΔpH). All three components are tethered by the Nernst equation, Δp = (ΔΨ) − 2.303RT/nF(ΔpH)^12^. Studying the effect of CPF on DiSC_3_(5) fluorescence, which localizes in the bacterial membrane in a ΔΨ dependent manner, illustrated the action of CPF on membrane energy. Membrane localization of DiSC_3_(5) decreases its fluorescence and PMF dissipation releases DiSC_3_(5) from membrane to media and increases its fluorescence^13^. DiSC_3_(5) fluorescence increased upon exposure to CPF (Fig. 2a) and the increase in fluorescence was proportional to the concentration of CPF (Fig. 2b). A similar result was observed when DiSC_3_(5)-labelled cells were exposed to a known proton translocator, carbonyl cyanide 3-chlorophenylhydrazone (CCCP), whereas no change in fluorescence was observed in the control sample (Fig. 2a). These observations indicate that CPF dissipates the ΔΨ of the cytoplasmic membrane.

### CPF acidifies the cellular cytoplasm

**Figure 3:**
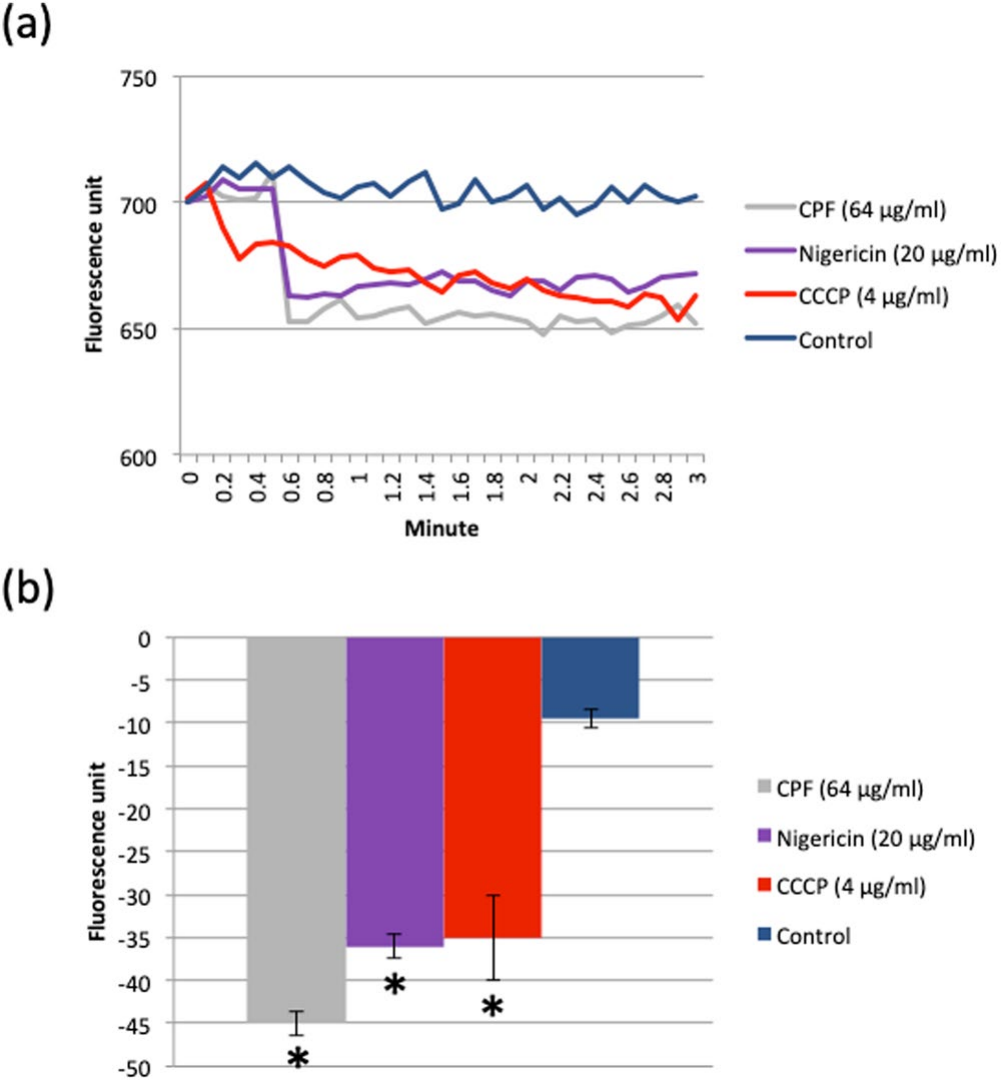
CPF carries proton. (**a**) BCECF fluorescence spectra and the effects of individual exposure to CPF, nigericin or CCCP on BCECF fluorescence. (**a**) BCECF fluorescence changes as a result of CPF, nigericin or CCCP exposure. A two-tailed t-test between control and samples was performed, significant difference with p < 0.05 is highlighted by asterisk.

The effects of CPF on ΔpH change with the dissipation of the ΔΨ upon CPF exposure was studied using a cytoplasm specific fluorophore, 2′,7′-Bis(2-carboxyethyl)-5(6)-carboxyfluorescein acetoxymethylester (BCECF-AM). It is known that upon entering into the cytoplasm BCECF-AM is hydrolysed by a cytoplasmic esterase to produce the BCECF fluorophore^14^. The fluorescence of BCECF is dependent on the pH of the cytoplasm. While under normal conditions the cytoplasmic pH is alkaline and BCECF fluorescence is high, a reduction in the fluorescence signal is expected following acidification of the cytoplasm due to influx of H^+^ via proton translocation^15^. A marked reduction in BCECF fluorescence was observed upon exposure to CPF or known proton translocators such as nigericin and CCCP (Fig. 3a). The cytoplasmic acidification induced by CPF was comparable to those caused by nigericin and CCCP (Fig. 3b).

### Co-administration of DOX with CPF alters the CPF-dependent gene expression programme

A total of 1594 genes changed expression profile at the transcript level upon exposure to CPF/DOX. Of these, 1058 genes were affected after 30 min and 1440 after 90 min with 903 genes being common to both time points (Table 1). Using the interaction Eq. (1) (see Material and Methods), we identified 485 genes with an interaction fold change (FC_I_) value significantly different from the sum of FCs for CPF and DOX separately (Supplementary Table S1). After 90 min exposure, 480 genes were interactively regulated, with 20 of these also identified at the 30 min time point. Only five genes were exclusively regulated after 30 min (phosphoglyceromutase, *gpmI*; triosephosphate isomerase, *tpi*; phosphoglycerate kinase, *pgk*; central glycolytic genes regulator, *cggR*; phosphoribosylformylglycinamidine synthase, UH47_09860). These were all upregulated and 4 out of 5 have predicted roles in glycolysis/gluconeogenesis (Table 2).

At the protein level, 60 genes were identified using the interaction formula analysis and 15 of them overlapped with the transcriptomic results. Two genes, pyridoxal biosynthesis protein (*pdxS*) and serine hydroxymethyltransferase (*glyA*), were regulated with unmatching direction, i.e. they were upregulated at the transcriptomic level but downregulated at the proteomic level. The complete list of interactively regulated genes is shown in Table S1.

Two types of interactive regulation were observed: FC_CPF/DOX_ and FC_I_ values had either the same (type 1) or the opposite (type 2) regulation direction. Several steps of the TCA cycle were interactively downregulated (*gltA*, *icd*, *acnA*, *fum*, *sdhC*). This pathway was downregulated by DOX, whereas it was upregulated by CPF alone with even higher absolute FC. Despite this upregulation, values calculated for both FC_CPF/DOX_ and FC_I_ indicated downregulation, indicating that both drugs together potentiate the effect of DOX instead of neutralising each other (type 1 interactive regulation).

Importantly, *sucD* generating ATP and malate dehydrogenase (*mqo*) producing NADH were interactively downregulated, as detected at the protein level. Moreover, several additional TCA enzymes up- or unregulated by CPF were interactively downregulated (*sucA*, *sucB*, *sucC, sdhA*, *sdhB*). Another important finding is that expression of ATP synthase, that was uninfluenced under exposure to CPF, was found to decrease when CPF and DOX were used in combination. We detected downregulation of three subunits of F_0_ (A,B,C) and F_1_ (β, ε, interactive: α, γ, σ) responsible for generating ATP. Moreover, two enzymes (dihydroxynaphthoic acid synthetase, *menB*; 2-succinyl-6-hydroxy-2, 4-cyclohexadiene-1-carboxylate synthase, *menH*) in the biosynthesis pathway of menaquinone (the electron carrier in respiratory chain) were also downregulated.

Pyruvate dehydrogenase (*pdhA*) unregulated by CPF was also interactively downregulated. The AckA protein, which activates acetate for conversion to pyruvate, interactively decreased in abundance as detected by proteomics. Almost all alternative carbon source genes (except the gene for melobiose) were very weakly or not upregulated by CPF/DOX despite being very highly upregulated by each drug individually, thus resulting in a negative FC_I_ (type 2 interactive regulation).

One of the strongest interactively downregulated pathways was tryptophan synthesis from chorismate (detected for *trpABCDGF* and *trpA* on protein level). Arginine conversion pathways (including *gdhA*, *rocD*; 1-pyrroline-5-carboxylate dehydrogenase, UH47_09200 and glutamate-1-semialdehyde aminotransferase, *hemL*) and peptide ABC transporter consisting of subunits UH47_06450 and UH47_06455 were also interactively downregulated. Importantly, carbamate kinase (*arcC*), which generates ATP molecule during arginine utilisation, was interactively downregulated on the protein level in addition to *arcA* and *arcB* downregulated on transcript level. Proteomics analysis also showed interactive downregulation of various proteins related to stress responses (protease, *clpP*; catalase, *katE;* ribosome-associated translation inhibitor, *raiA*; alkaline shock protein 23, UH47_11605; universal stress protein, UH47_02170; *phoP* family transcriptional regulator, *phoB*). UH47_02170, *phoP* and *phoB* were also downregulated at the transcriptional level.

Proteomic analysis revealed that elongation factor Tu (*tuf*), which was downregulated by DOX (Supplementary Table S1), was even more downregulated by the combination of the two drugs. Only one ribosomal subunit (30S S2, *rpsB*) was detected as being interactively downregulated. In this case both CPF and DOX upregulated this subunit and also FC_CPF/DOX_ was positive but not so high as it would be expected after summing the effects of each drug (type 2 interactive regulation). Most of the ribosomal subunits (30S S6, *rpsF*; 50S L7/L12, *rplL*; 50S L25, *rplY*; 50S L10, *rplJ*) detected by proteomics had positive FC_CPF/DOX_ and FC_I_ values. We also found three interactively upregulated tRNA ligase genes (*valS*, *alaS*, UH47_11775).

CPF and DOX in combination interactively increased expression of *tetK* and interestingly also the TetR family transcriptional regulator (UH47_08515), whereas sub-inhibitory concentration of DOX alone did not affect expression of these genes and CPF downregulated both (Table 2).

### CPF and DOX synergistically inhibit protein but not DNA synthesis

**Figure 4:**
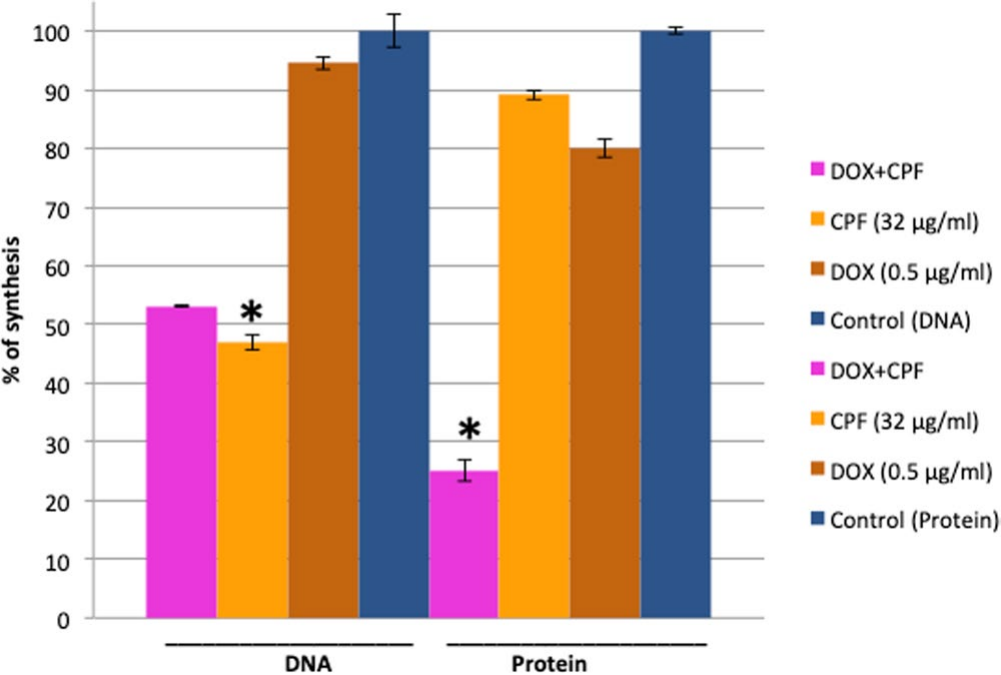
CPF-DOX combination inhibits protein synthesis synergistically. Percentages of radiolabelled DNA and protein precursors incorporation compared to unexposed control are presented as average values of two individual measurements. DNA and protein synthesis panels are designated by line and labelled as DNA and Protein. A two-tailed t-test between control and samples was performed, significant difference with p < 0.05 is highlighted by asterisk.

Macromolecule biosynthesis assays using radiolabelled ^3^H-Thymidine (DNA precursor) and ^3^H-leucine (protein precursor) were performed to measure the rate of DNA and protein synthesis in the presence of CPF and DOX, used individually and in combination. A 50% reduction in the rate of DNA synthesis was observed when cells were exposed to 32 µg/ml of CPF (Fig. 4). No marked change in the rate of DNA synthesis was observed when cells were exposed to 0.5 µg/ml of DOX. The same concentration of DOX did not show any additional effect on DNA synthesis when cells were exposed to the two drugs in combination. The rate of protein synthesis was reduced by only 20% and 10% upon exposure to DOX and CPF alone, respectively, whereas a reduction of 75% was observed following simultaneous exposure to both drugs (Fig. 4).

## Discussion

### CPF is a proton translocator

The CPF-dependent changes in gene expression identified using transcriptomics and proteomics are consistent with a role for CPF in compromising membrane bioenergetics. This hypothesis was confirmed by our biophysical experiments, which demonstrated that CPF acts as a proton translocator. This conclusion is based upon three observations when TetK-positive MRSP are exposed to CPF: i) the high EtBr incorporation showing activity of CPF on the cytoplasmic membrane, ii) the DiSC_3_(5) fluorescence increment documenting its ionophore activity, and iii) the BCECF fluorescence drop supporting its proton carrier activity. The ability of CPF to cause proton translocation across cellular membranes is also consistent with the recent finding that this drug causes an oxidative stress response in the mitochondria of the canine dog mucosal cells^16^. Mitochondrial and bacterial membranes are known to be similar^17^. Compared to CCCP, CPF is a weaker dissipator of PMF since a similar ΔpH dissipation was obtained by exposure to 20 µM of CCCP and 234 µM of CPF (64 µg/ml), indicating that CCCP is almost ten times more efficient as a proton carrier compared to CPF. The proton translocation function of CCCP is due to its weak acid property^18^ and the chemical structure of CPF suggests that the mechanism involved could be the same, even though the number of proton carrying moieties in CPF is lower compared to CCCP (Supplementary Figure S2).

### Metabolic compensation strategy for CPF-induced PMF depletion

The potential energy of the membrane PMF is used for the synthesis of ATP in the process of oxidative phosphorylation. This process is much more efficient than substrate phosphorylation, which is only a secondary process to gain ATP^19^. Muthaiyan *et. al*.^20^ reported that *S. aureus* strongly upregulates expression of all genes exclusively encoding glycolytic enzymes (with no impact on gluconeogenesis) to compensate for depletion of PMF after 15 min of exposure to CCCP. We did not observe this type of compensation after 30 and 90 min exposure to CPF. In contrast to *S. aureus*, *S. pseudintermedius* does not possess an enzymatic system to hydrolyse and metabolise starch (according to KEGG PATHWAY Database)^21^, the main carbohydrate source in cation adjusted Mueller-Hinton broth (CAMHB), the medium used in both studies. It can be hypothesised that during growth in CAMHB, MRSP first metabolises the residual glucose present in the formulation before switching to peptides/amino acids. After 90 min exposure to CPF, when residual glucose was exploited, we observed a pattern of gene regulation typical of inactivation of carbon catabolite repression^22^. This included downregulation of glucose uptake and upregulation of several alternative carbohydrate carbon source uptake and activation systems. Glycolytic substrate phosphorylation can run on alternative carbon compounds^23,24^ but this was evidently not a primary energy compensation strategy because it would require activation of fermentation to regenerate NAD^+^, which was not observed. Downregulation of two glycolytic enzymes and upregulation of only one enzyme that increases the pool of NADH supports this proposal. Instead, we observed an increase in reactions and transport processes providing substrates for, and regenerating intermediates of, the TCA cycle, i.e. pyruvate from lactate, acetyl-CoA from acetate, acetonin and most significantly from lipid degradation, oxoglutarate from histidine (and probably other amino acids since we observed upregulation of many peptide transporters, oligo- and aminopeptidases, and CAMHB medium is rich in peptides); and succinate, fumurate and malate via the C4-dicarboxylate transporter. We also detected very consistent upregulation of the TCA cycle. This matches the observed upregulation of respiratory complex I and II. In some organisms, the activity of chain components can also be allosterically inhibited by ATP^25^ that is presumably reduced under CPF exposure. Altogether, these findings suggest that MRSP attempts to compensate for the proton translocation mediated by CPF by sending protons from overproduced NADH to the extracellular space.

Interestingly, we found that the arginine utilisation pathway was notably upregulated in response to CPF. Arginine can be generated from increased octopine degradation^26^ and from putrescine since its uptake was also upregulated. Energy depletion and arginine upregulate the catabolic ADI pathway and that provides ATP^27^. Many bacteria exploit arginine as an energy source by this pathway^28,29^. Thus, upregulation of this pathway could also be an alternative energy strategy used by MRSP to compensate for the action of CPF.

### CPF acts in synergy with DOX to inhibit protein synthesis

CPF induces synergistic protein synthesis inhibition in combination with DOX in *tetK-*mediated DOX-resistant MRSP ST71 (Fig. 4). This suggests that cytoplasmic levels of DOX, which depend on the activity of the TetK efflux pump, increase in the presence of CPF. The TetK pump exports DOX out of cells in exchange for H^+^ ions^30,31^. Hence, the most likely mechanism behind the observed CPF/DOX synergy in *tetK*-mediated DOX-resistant strains is that CPF’s PMF dissipation inactivates the TetK efflux pump and indirectly increases the cytoplasmic levels of DOX, which in turn inhibits protein synthesis and cell growth. Proteomics analysis revealed decreased expression of RaiA, a ribosome-associated translation inhibitor, whereas expression of many ribosomal subunits increased under exposure to the drugs in combination. The latter could be a consequence of the loss of DOX-inhibited ribosomal subunits.

The PMF dissipation action of CPF inactivates the TetK efflux pump (the cause of DOX resistance), which indirectly increases the cytoplasmic DOX levels and consequently inhibition of protein synthesis. Nevertheless, reduction of ΔpH by CPF simultaneously reduces the pH gradient dependent diffusion of DOX into the cytoplasm since cellular uptake of DOX depends on the ΔpH of the cytoplasmic membrane^32^. Thus, CPF-mediated PMF dissipation has both positive and negative effects on the antimicrobial activity of DOX. Simultaneously it inhibits the TetK efflux pump (positive effect) and reduces the diffusion of DOX inside the cell (negative effect). This explains the moderate synergy (ΣFICI = 0.325) that we observed between DOX and CPF in our previous study^18^.

### Interference between CPF and DOX pathways abrogates compensation of PMF depletion

**Figure 5:**
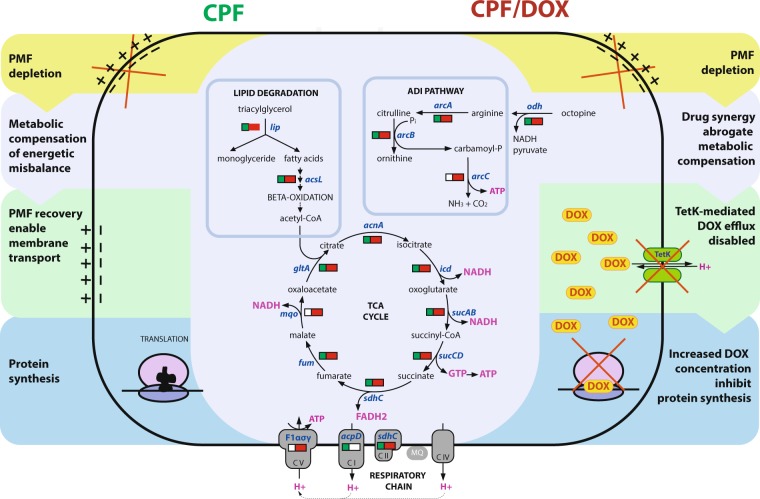
Proposed mechanism of action of CPF alone and in combination with DOX. The figure illustrates the proposed mechanism of action of CPF alone (left) and in combination with DOX (right), including the most consistently regulated pathways related with energy metabolism. The bars indicate a gene regulation mode in the drawn metabolic pathways. They contain square (left) representing CPF and rectangle (right) representing CPF/DOX interactions. Green in bars indicates upregulations and red upregulation.

Transcriptomic and proteomic profiling clearly showed that presence of DOX, even at a sub-inhibitory concentration, substantially interferes with the metabolic strategy to restore energetic balance in the presence of CPF. CPF regulates only 12% of the genes of the genome. The number of genes regulated by DOX is close to the number of genes regulated by CPF/DOX used in combination, approximately 60%. Despite this, DOX has limited effects on the growth of MRSP. However, when MRSP is exposed to the two drugs in combination, the crosstalk between these unmanifested (in regards to viability) alterations with metabolic adaptation to CPF is fatal for the bacteria. Most importantly, (I) the interactions are strongly reflected in downregulation of the entire TCA cycle that is the core of carbon metabolism. Consequently, the influx of TCA substrate acetyl-CoA from many reactions as well as regeneration of TCA intermediates mainly by lipid and histidine degradation were either not upregulated in comparison to CPF exposure alone or interactively downregulated. (II). The drug interactions cause downregulation of all subunits of ATP synthase. By these two alterations in metabolic adaptation to CPF bacterial cells lose the opportunity to overproduce NADH and ATP to compensate for PMF depletion. This is supported by the fact that the phosphate starvation regulator PhoP, which is normally expressed in response to a lack of inorganic phosphate^33^, was downregulated following exposure to CPF and DOX. Since ATP production decreases, there is no inorganic phosphate deficit. The mechanism proposed to explain the action of CPF and the synergy between CPF and DOX is illustrated in Fig. 5.

## Conclusions

This study demonstrates that CPF is a proton translocator that paralyzes PMF-dependent efflux of DOX mediated by TetK, thereby allowing intracellular DOX concentrations that are required for inhibition of protein synthesis. PMF depletion induced by CPF challenges oxidative phosphorylation, while energy balance is sustained by metabolic adaptations. Interference between CPF and DOX pathway alterations abrogates this energetic compensation.

## Materials and Methods

### Bacterial strain

MRSP ST71 E104^3^ was used as a model strain for investigating the mechanism of synergy between CPF and DOX. Genomic DNA extraction was performed using MasterPure™ Gram Positive DNA Purification Kit (Epicenter). A *de novo* whole genome sequencing library was created using the Nextera XT DNA Library Preparation Kit (Illumina). The library was run with a 250-bp paired-end read module using the MiSeq platform. The average depth of sequencing coverage was 121. The reference genome was annotated using the Prokaryotic Genomes Automatic Annotation Pipeline (PGAAP)^34^ developed by the National Center for Biotechnology Information (NCBI). The reference genome sequence for MRSP E104 is available at NCBI under the accession number: LAWU00000000. The transcriptome and proteome analyse is based on the annotations available for this genome on 05 July 2015.

### Culture conditions and experimental design for protein and RNA sampling

Overnight culture was diluted to an OD_600_ of 0.05 in CAMHB and re-grown to OD_600_ 0.4 (10^8^ CFU/ml) in the same medium. A main culture was initiated by diluting this pre-culture 1:1000 (10^5^ CFU/ml) in three replicate flasks (5 L, 1050 ml in each). At an OD_600_ of 0.1 (10^7^ CFU/ml) every replicate culture was split into set of four flasks (1 L, 250 ml in each). Three cultures in each set were exposed to DOX (0.5 µg/ml), CPF (32 µg/ml) or both compounds (0.5/32 µg/ml) and further incubated with an untreated control. As CPF was used as a dimethylsulfoxide (DMSO) solution, all cultures including controls were adjusted to have an equal final DMSO concentration (≤0.4%). OD_600_ of all cultures was measured every hour for the next 12 h. All cultures were incubated at 37 °C and 180 rpm. 45 ml aliquots of each culture were collected after 90 min of exposure, pelleted and washed once with saline. Cultivation and treatment procedures were reproduced for RNA extraction. 6–9 ml aliquots of each culture were collected after 30 and 90 min of exposure. Samples were immediately suspended in RNA Protect solution (twice the amount of culture, QIAGEN), incubated for 5 min and centrifuged at 4000 rpm for 10 min at room temperature. Supplementary Figure S1 summarise experimental plan for the expression study including sample numbers, exposure times and conditions.

### RNA sequencing and read mapping

Total RNA was extracted from MRSP E104 cells using an RNeasy Mini Kit (Qiagen) by adapting the manufacturer’s instructions to own settings. Cells were lysed in the presence of 300 mg acid-washed glass beads (150–600 μm diameter, Sigma) per sample using a Fast Prep Lyser (Eppendorf) with three cycles of 1 min at maximum speed and one cycle of 7 min, cooling the samples on ice for 2 min before and between cycles. The cooling on ice was used to prevent unwanted heating during RNA extraction. On column DNA digestion was performed using the RNase-Free DNase Set kit (Qiagen). The integrity of the RNA after isolation was verified using a NanoDrop^TM^ (absorbance ratios 260/280 nm and 260/230 nm ≥ 1.8) and a Bioanalyzer 2100 (RNA Integrity Number (RIN) ≥ 7). Samples of total RNA (≥5 µg, measured on a Qubit) were submitted for transcriptome sequencing at a commercial provider (Beijing Genomics Institute, BGI) using an Illumina HiSeq. 2000 machine with TruSeq V3 sequencing kits. Prior to sequencing, all samples were treated with DNaseI (New England Biolabs® Inc) and then enriched for mRNA molecules via ribosome-depletion using the Ribo-Zero™ magnetic Kit for bacteria (Epicentre). A quality control of the reads was done by a standard procedure performed at BGI using the software SOAPnuke^35^ and confirmed by the FastQC software (https://www.bioinformatics.babraham.ac.uk/projects/fastqc/). The reference genome sequences of *S. pseudintermedius* strain E104 is available at NCBI under the accession numbers: LAWU00000000. The RNA-sequencing reads are available in the Short Read Archive (SRA) at NCBI under the accession numbers: SRX3206467 to SRX3206493. Library construction was done using a prokaryotic transcriptome library construction protocol (developed by BGI). Sequenced reads (>10 million paired-end 90 nt reads per sample) were mapped to the reference genome using TopHat v2.0.14^36^ employing the default settings but with splice awareness turned off. Mapped reads were processed and analysed in R^37^. Reads mapping to annotated genome features were counted using Rsubread^38^, disallowing duplicate reads and reads that map to more than one location. Reads overlapping adjacent genes were counted for each gene. Genes exhibiting generally low transcript counts were filtered out from the downstream analysis (9 genes removed), retaining transcripts with > 0.5 counts per million (cpm) in at least 3 of the samples.

### Protein extraction and two-dimensional differential in-gel electrophoresis (2D DIGE)

E104 cells were lysed by lysostaphin (30 min at 37 °C) and three rounds of liquid nitrogen frozen/thawed in presence of protease inhibitor (GE Life Sciences, as all following reagents, kits, devices and software in this paragraph). Lysates were treated with nuclease (40 min at room temperature). Next, proteins were purified via 2D Clean-up Kit, solubilized in DIGE buffer (7 M urea, 2 M thiourea, 4% CHAPS, and 30 mM Tris-HCl) and protein concentrations determined using the 2D Quant Kit. The CyDye DIGE Fluor minimal dyes, Cy3 or Cy5, were used to label all treated samples and untreated control (50 μg of protein each) according to Ettan DIGE System manual. To eliminate dye-specific bias, all types of samples were dye-swapped by labelling with both Cy3 and Cy5. Internal standard was prepared by mixing aliquots of every protein sample (25 μg from each) and labelled with Cy2. Protein samples were combined in pairs and mixed together with an aliquot of internal standard, and supplemented with 1% dithiothreitol, 1% IPG buffer pH 4–7 and 0.02% bromophenol blue.

Prior to isoelectric focusing (IEF), IPG strips (pH 4–7, 24 cm) were rehydrated in DeStreak rehydration solution with 0.5% IPG buffer pH 4–7 overnight. The samples were applied using the cup-loading method and IEF was performed at a total of 50 kVh per stripe on Ettan IPGphor. IPG strips were subsequently reduced, alkylated (15 min each) and proteins were separated in the second dimension with 12.5% SDS-PAGE gels on Ettan DALT six Electrophoresis Unit according to manual. Immediately after the second dimension, gels were imaged at excitation/emission wavelengths of Cy2 (488/520 nm), Cy3 (532/580 nm), and Cy5 (633/670 nm) at 100 μm resolution by using Typhoon 9500 Variable Mode Imager. The obtained images were exported as 16-bit GEL files. Spots on separate gels were detected and analysed using Difference In-gel Analysis (DIA) module and matched between gels using BVA module within DeCyder Image Analysis Software v 7.0 software. Comprehensive Ettan DIGE System user manual can be accessed on https://www.mcgill.ca/cian/files/cian/ge_dige_manual.pdf.

### Protein identification

Prior to spot identification, gels were stained with Sypro Ruby (Sigma-Aldricht) according to the manufacturer manual. Selected spots qualified based on interaction formula described in next paragraph were manually excised and subjected to in-gel digestion using Trypsin (Sigma-Aldricht) following the manufacturer’s instructions. Next, peptides were isolated from gel spots (by H_2_O, trifluoroacetic acid and acetonitrile solutions) and purified by RP C_18_ chromatography (μZipTip, Millipore). Peptides extracted from protein spots were analysed by an EASY-nLC mounted with an EASY-Spray column (PepMap, C_18_, 3μm, 100 Å, 75 μm × 15 cm) coupled with a Q Exactive Biotech mass spectrometer (both Thermo Fisher Scientific). Buffer A consisted of 0.1% formic acid (FA) in water, and buffer B was 0.1% FA in 99% acetonitrile. 5 µl of sample were injected. The flowrate was 100 nl/min and the gradient consisted of linear increase from 0% to 30% B in 30 min. On-line MS/MS spectra were recorded in the positive mode using the Full MS method with a resolution of 70000, an AGC target of 3e^6^, max IT of 50 ms and a mass range from 200 to 2000 m/z. The top 7 spectra from the MS were analysed using the dd-MS^2^ method, with a resolution of 35000 and AGC target of 1e^6^. Mass spectra of proteins were exported and compared to the whole MRSP E104 proteome using Proteome Discoverer software (v 1.4, Thermo Fisher Scientific). The search method used was Sequest HT, with precursor mass tolerance 10 ppm and fragment mass tolerance set to 0.05 Da, static modification carbamidomethyl + 57.021 Da for cysteine. The target Decoy PSM validator was used as validation method. The top score identified proteins for every spot were selected. Identification details for selected protein spots are listed in Supplementary Table S2. The MS data and the preparative gel picture have been deposited to the ProteomeXchange Consortium via the PRIDE^38^ repository with the dataset identifier PXD013725.

### Analysis of gene expression data

Normalized values for transcript and protein abundance in the log2 scale were obtained from the transcriptomic data by Trimmed Mean of M values (TMM) normalization^39,40^ and from the proteomic data by transforming protein pick volumes using Decyder. Normalized values for all individual exposure conditions (DOX, CPF, DOX-CPF) were tested for differential expression by comparing against the control (no exposure) sample taken at the corresponding time point. Testing was performed in R using LIMMA linear modelling, applying the LIMMA voom method^40^ to both the RNA-seq and 2D DIGE data, and calculating adjusted P-values using the Benjamini and Hochberg multiple testing correction. In order to identify changes in gene expression associated with the synergistic interaction of CPF and DOX, a null hypothesis based on a multiplicative interaction model^41^ was assumed. Thus, for example, a transcript or protein that exhibits a 2-fold reduction in abundance following CPF exposure and also a 2-fold reduction following DOX exposure, is expected to show a 4-fold reduction following exposure with both CPF and DOX in combination. In the log scale, this can be represented by the following Eq. (1), where NA corresponds to normalized abundance:

1 $$({\mathrm{Log}}_{2}{{\rm{NA}}}_{{\rm{CPF}}-{\rm{DOX}}}-{\mathrm{Log}}_{2}{{\rm{NA}}}_{{\rm{Control}}})-(({\mathrm{Log}}_{2}{{\rm{NA}}}_{{\rm{DOX}}}-{\mathrm{Log}}_{2}{{\rm{NA}}}_{{\rm{Control}}})+({\mathrm{Log}}_{2}{{\rm{NA}}}_{{\rm{CPF}}}-{\mathrm{Log}}_{2}{{\rm{NA}}}_{{\rm{Control}}}))=0$$

Significant deviations from the null hypothesis are represented by an interaction score Log_2_FC_I_ calculated using the same equation (where Log_2_FC_I_ = 0 indicates no interaction), and are defined by an adjusted P-value ≤ 0.05 and Log_2_FC_I_ ≥ 1 or ≤ −1 (2-fold change limit) for transcript abundance, and an adjusted P-value ≤ 0.05 and Log_2_FC_I_ ≥ 0.585 or ≤ −0.585 (1.5-fold change limit) for protein abundance.

### Protein annotation and pathway prediction

Altered pathway prediction for differentially expressed genes as an input was performed with support of geneGO^42^ annotations, Kyoto Encyclopedia of Genes and Genomes (KEGG_PATHWAY)^21^, BioCyc^43^ and String^44^ However, due to weak NCBI annotation application of the listed bioinformatics tools for *S. pseudintermedius* genomes, most of the annotation and pathway reconstruction was performed or refined manually by BLAST analysis and literature searches based on closely related species.

### Measurement of EtBr incorporation rate

Measurement of EtBr incorporation rates was performed following a modified protocol from Bulathsinghala *et al*.^45^. Briefly, overnight culture of E104 was diluted 1:100 in CAMHB (Sigma-Aldrich) and sub-cultured to reach an OD_600_ of 0.2. The culture was exposed to different concentrations of CPF (16, 32 or 64 μg/ml) for 5 min at room temperature before transferring into a cuvette and adding 10 µg/ml of EtBr. The EtBr fluorescence spectra were recorded in PerkinElmer LS50B fluorescence spectrometer at Excitation/Emission (Ex/Em) wavelengths 520 nm/590 nm for 3 min using time drive application of FLWINLAB software. Finally, EtBr fluorescence spectra over time were plotted as arbitrary fluorescence unit (FU). Linear fluorescence increment in initial 30 seconds (FU_30sec_ - FU_0sec_) was multiplied by 2 to calculate the rate of fluorescence increment per minute (FU/min). Two-tailed t-test was used to detect significant differences between CPF-exposed and control samples. All probe-based experiments were performed with minimum two technical and two biological replicates.

### Comparative study of bacterial membrane potential

A fresh culture of E104 was diluted to OD_600_ of 0.1 in CAMHB and cells were labelled with 1 µM DiSC_3_(5). The fluorescence spectra of labelled cells were taken at Ex/Em wavelengths 546 nm/573 nm as described previously^46^. After reading of initial stable DiSC_3_(5) spectra, labelled cells were treated with CPF (16, 32 or 64 μg/ml) or carbonyl cyanide 3-chlorophenylhydrazone (CCCP, 0.05, 0.1 or 0.2 µg/ml), a known proton translocator (positive control). Changes in DiSC_3_(5) fluorescence over time (FU_after treatment_ − FU_before treatment_) were recorded and plotted.

### Measurement of cytoplasmic pH change

E104 was cultured in presence of 10 µg/ml of BCECF-AM, a pH-sensitive cytoplasm-specific dye, for 30 min at 30 °C. Incubated cells were pelleted down and re-suspended in phosphate buffer saline supplemented with 25 mM glucose adjusting OD_600_ to 0.2. The cell suspension was kept at room temperature for 1 hour to equilibrate the cytoplasmic pH and finally BCECF fluorescence was recorded at Ex/Em wavelengths 485 nm/520 nm. Nigericin and CCCP were used as positive controls in view of their known activity as proton translocators. After initial stable reading of BCECF spectra, labelled cells were treated with CPF (64 μg/ml), nigericin (20 μg/ml) and CCCP (4 μg/ml) individually, and changes in BCECF fluorescence between the time points 0.2 and 2.8 mins (FU_2.8 min_ – FU_0.2 min_) were recorded and plotted.

### Determination of macromolecule biosynthesis rates

Overnight E104 culture was diluted 1:100 in minimal medium (0.02 M HEPES, 0.002 M MgSO_4_, 0.0001 M CaCl_2_, 0.4% succinic acid, 0.043 M NaCl_2_, 0.5% (NH4)_2_SO4) supplemented with 5% tryptic soy broth (TSB) and sub-cultured to OD_600_ of 0.2. Grown cells were pelleted down by centrifugation and re-suspended in fresh minimal media followed by incubation with either DOX (0.5 µg/ml), CPF (32 µg/ml) or DOX/CPF combination (0.5/32 µg/ml) for 15 min. Incubated cells were pulse labelled for 20 min with 25 µl of radiolabelled DNA or protein synthesis precursors, (50 µCi) ^3^H-Thymidine or ^3^H-leucine (Ultima Gold, PerkinElmer), respectively. Finally, radiolabelled cells were precipitated with equal volume of 30% trichloroacetic acid (TCA) as described previously^13,46^. Precipitates were filtered on cellulose membrane (cat no. AAWG0250C, Millipore) and washed by two sequential washes of ice-cold 15% TCA and two washes of ice-cold water. Filters were dried and stored in scintillation vials. 3 ml scintillation fluid was added to each vial and ^3^H count was taken in Beckman Coulter LS6500 liquid scintillation counter for 1 min. Macromolecule biosynthesis rates were calculated based on the assumption that the rates of DNA and protein precursor incorporation were 100% in the control sample.

## Supplementary information

Supplementary Information

Supplementary Information

Supplementary Information
