# Supplementary material for: Carprofen-induced depletion of proton motive force reverses TetK-mediated doxycycline resistance in methicillin-resistant Staphylococcus pseudintermedius
Source: Sci Rep. 2019 Nov 28;9:17834. doi: 10.1038/s41598-019-54091-4 (PMC6882848; doi:10.1038/s41598-019-54091-4)
Supplement: Supplementary file 1 — Supplementary Information [file 41598_2019_54091_MOESM1_ESM.pdf]

Carprofen-induced depletion of proton motive force reverses TetK-mediated doxycycline resistance in methicillin-resistant *Staphylococcus pseudintermedius*

Zofia Magnowska[1a*], Bimal Jana[1a], Rikke Prejh Brochmann[1a], Andrew Hesketh[2,3], Rene Lametsch[4], Cristian De Gobba[4] and Luca Guardabassi[1,5*]

1 Department of Veterinary and Animal Sciences, Faculty of Health and Medical Sciences, University of Copenhagen, Frederiksberg, Denmark
2 Department of Biochemistry and Cambridge Systems Biology Centre, University of Cambridge, Cambridge, United Kingdom
3 School of Pharmacy and Biomolecular Sciences, University of Brighton, Brighton, United Kingdom
4 Department of Food Science, Faculty of Sciences, University of Copenhagen, Frederiksberg, Denmark
5 Department of Pathobiology and Population Sciences, The Royal Veterinary College, Hatfield, United Kingdom

[a] The first three authors equally contributed to the work

** Corresponding authors:*
Luca Guardabassi, e-mail: lg@sund.ku.dk
Zofia Magnowska, e-mail: zofia@sund.ku.dk

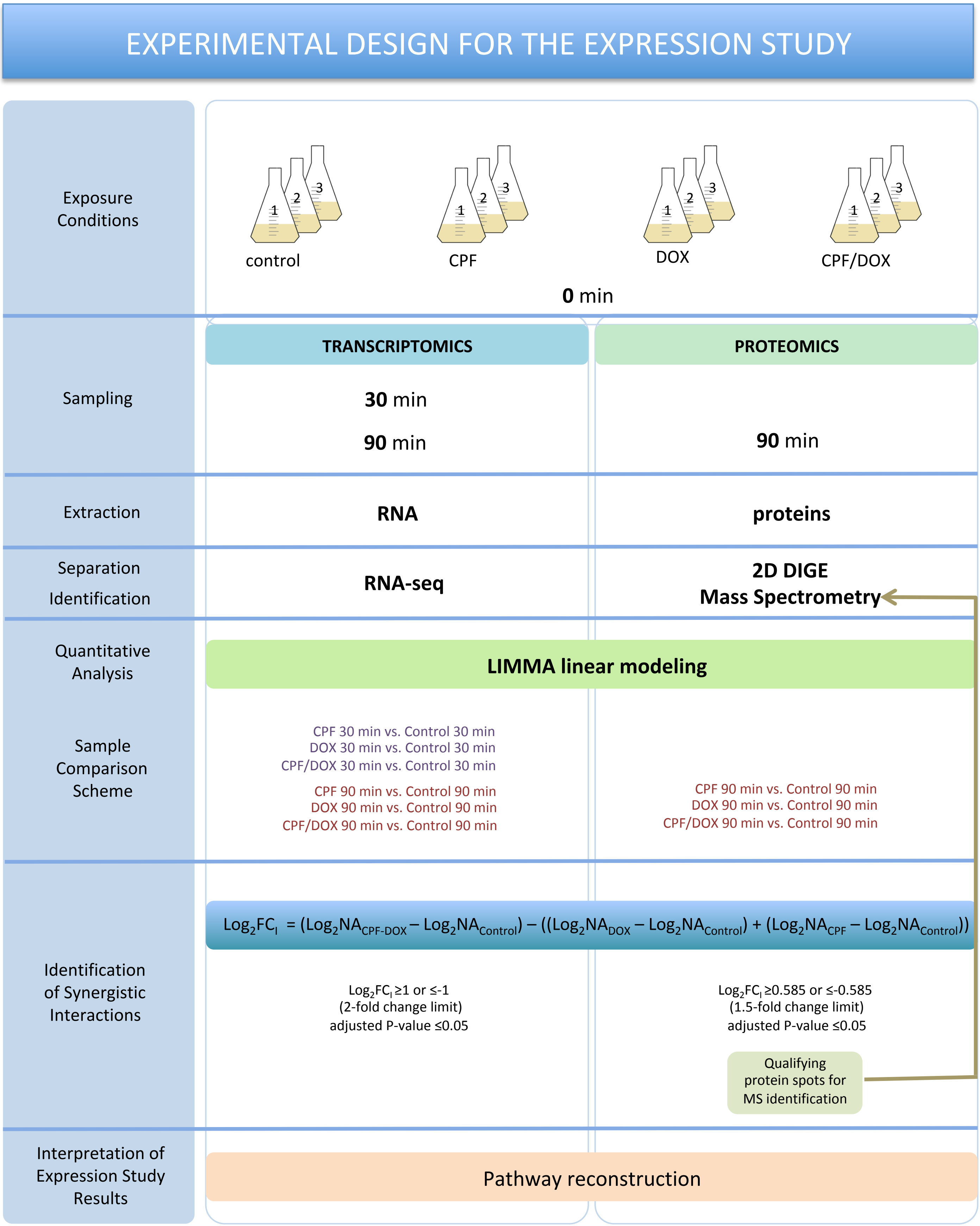

Supplementary Figure S1. Experimental design for the expression study.

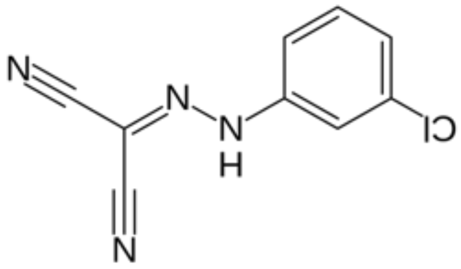

CCCP

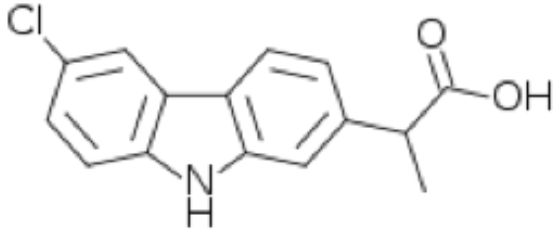

Carprofen

Supplementary Figure S2. Chemical formula of CCCP and CPF.
